# Prediction of Clinical Outcome for High-Intensity Focused Ultrasound Ablation of Uterine Leiomyomas Using Multiparametric MRI Radiomics-Based Machine Leaning Model

**DOI:** 10.3389/fonc.2021.618604

**Published:** 2021-09-10

**Authors:** Yineng Zheng, Liping Chen, Mengqi Liu, Jiahui Wu, Renqiang Yu, Fajin Lv

**Affiliations:** ^1^Department of Radiology, The First Affiliated Hospital of Chongqing Medical University, Chongqing, China; ^2^State Key Laboratory of Ultrasound in Medicine and Engineering, Chongqing Medical University, Chongqing, China

**Keywords:** radiomics, machine learning, HIFU, uterine leiomyoma ablation, preoperative prediction

## Abstract

**Objectives:**

This study sought to develop a multiparametric MRI radiomics-based machine learning model for the preoperative prediction of clinical success for high-intensity-focused ultrasound (HIFU) ablation of uterine leiomyomas.

**Methods:**

One hundred and thirty patients who received HIFU ablation therapy for uterine leiomyomas were enrolled in this retrospective study. Radiomics features were extracted from T2-weighted (T2WI) image and ADC map derived from diffusion-weighted imaging (DWI). Three feature selection algorithms including least absolute shrinkage and selection operator (LASSO), recursive feature elimination (RFE), and ReliefF algorithm were used to select radiomics features, respectively, which were fed into four machine learning classifiers including k-nearest neighbors (KNN), logistic regression (LR), random forest (RF), and support vector machine (SVM) for the construction of outcome prediction models before HIFU treatment. The performance, predication ability, and clinical usefulness of these models were verified and evaluated using receiver operating characteristics (ROC), calibration, and decision curve analyses.

**Results:**

The radiomics analysis provided an effective preoperative prediction for HIFU ablation of uterine leiomyomas. Using SVM with ReliefF algorithm, the multiparametric MRI radiomics model showed the favorable performance with average accuracy of 0.849, sensitivity of 0.814, specificity of 0.896, positive predictive value (PPV) of 0.903, negative predictive value (NPV) of 0.823, and the area under the ROC curve (AUC) of 0.887 (95% CI = 0.848–0.939) in fivefold cross-validation, followed by RF with ReliefF. Calibration and decision curve analyses confirmed the potential of model in predication ability and clinical usefulness.

**Conclusions:**

The radiomics-based machine learning model can predict preoperatively HIFU ablation response for the patients with uterine leiomyomas and contribute to determining individual treatment strategies.

## Introduction

Uterine leiomyomas are benign smooth-muscle neoplasm of the uterus in women of reproductive age, with a high morbidity of more than 70%, and seriously makes the quality of life of patients worse or even affects fertility (1). When uterine leiomyomas are symptomatic and pharmacotherapy fails (2), the choice between hysteromyomectomy and hysterectomy treatment depends on female fertility needs (3, 4), which is also a common factor for surgical removal of the uterus. Recently, high-intensity focused ultrasound (HIFU) ablation has played a significant role and been a subject of interest in noninvasive treatment modality of thermal ablation (5). Compared with hysterectomy, the use of HIFU as a noninvasive intervention in abundant clinical trial has demonstrated the potential to ablate the uterine leiomyomas by selective tissue heating, which can significantly improve symptoms resulting from the uterine leiomyomas (6, 7). However, preoperative evaluation is a crucial factor for ensuring a high ablation rate of leiomyoma tissue as well as the success rate of HIFU treatment, so preoperative outcome prediction would better guide clinical decision making for therapeutic strategy (8, 9).

In clinical practice, magnetic resonance imaging (MRI) is commonly used for preoperative evaluation and response assessment before and after HIFU ablation treatment, respectively (10). Several studies have investigated the relationship between the degree of signal intensity on T2-weighted (T2WI) images and treatment outcome of HIFU ablation for uterine leiomyomas, but only showed a limited predictive power (11, 12).

Radiomics has emerged as a promising tool to provide quantitative biomarkers from routine multimodal radiological images that can help recognize imaging information linked with treatment outcome (13–15), by considering that the high-throughput extraction of images is beyond the capabilities of the naked eye in clinical application. Radiomics-based machine learning is quite rapidly gaining importance in the medical field (16). It tries to identify patterns in imaging data and provide decision support by connecting these patterns to treatment outcome, which can facilitate higher precision in diagnosis and prognosis (17, 18).

However, no studies reported the radiomics analysis of nonenhanced MRI for outcome prediction in HIFU ablation, and there is still a lack of machine learning approaches to automatically predict HIFU treatment outcome so as to guide patient selection. This study aims to develop and validate multiparametric MRI radiomics-based machine leaning model to preoperatively predict clinical outcome of HIFU ablation of uterine leiomyomas.

## Materials and Methods

**Figure 1:**
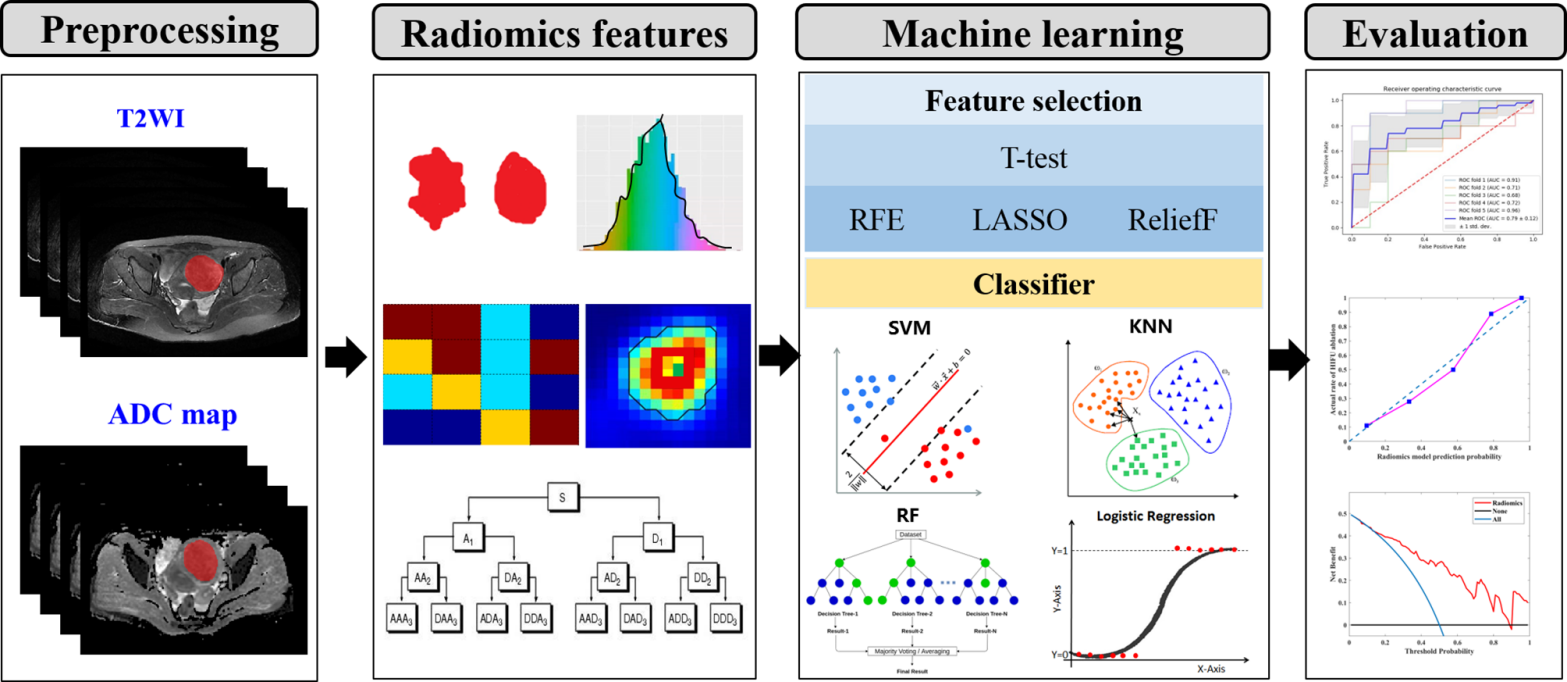
The conceptual flowchart of the present study. (I) lesion segmentation and preprocessing. (II) Quantitative radiomics features extraction. (III) Feature selection and classification. (IV) Performance evaluation of machine learning model. T2WI, T2-weighted imaging; RFE, recursive feature elimination; LASSO, least absolute shrinkage and selection operator; SVM, support vector machine; KNN, k-nearest neighbors; RF, random forest.

An overview of the proposed prediction model for the HIFU-based uterine leiomyoma ablation is illustrated in **Figure 1**. More details are given in the following sections.

### Study Population

**Figure 2:**
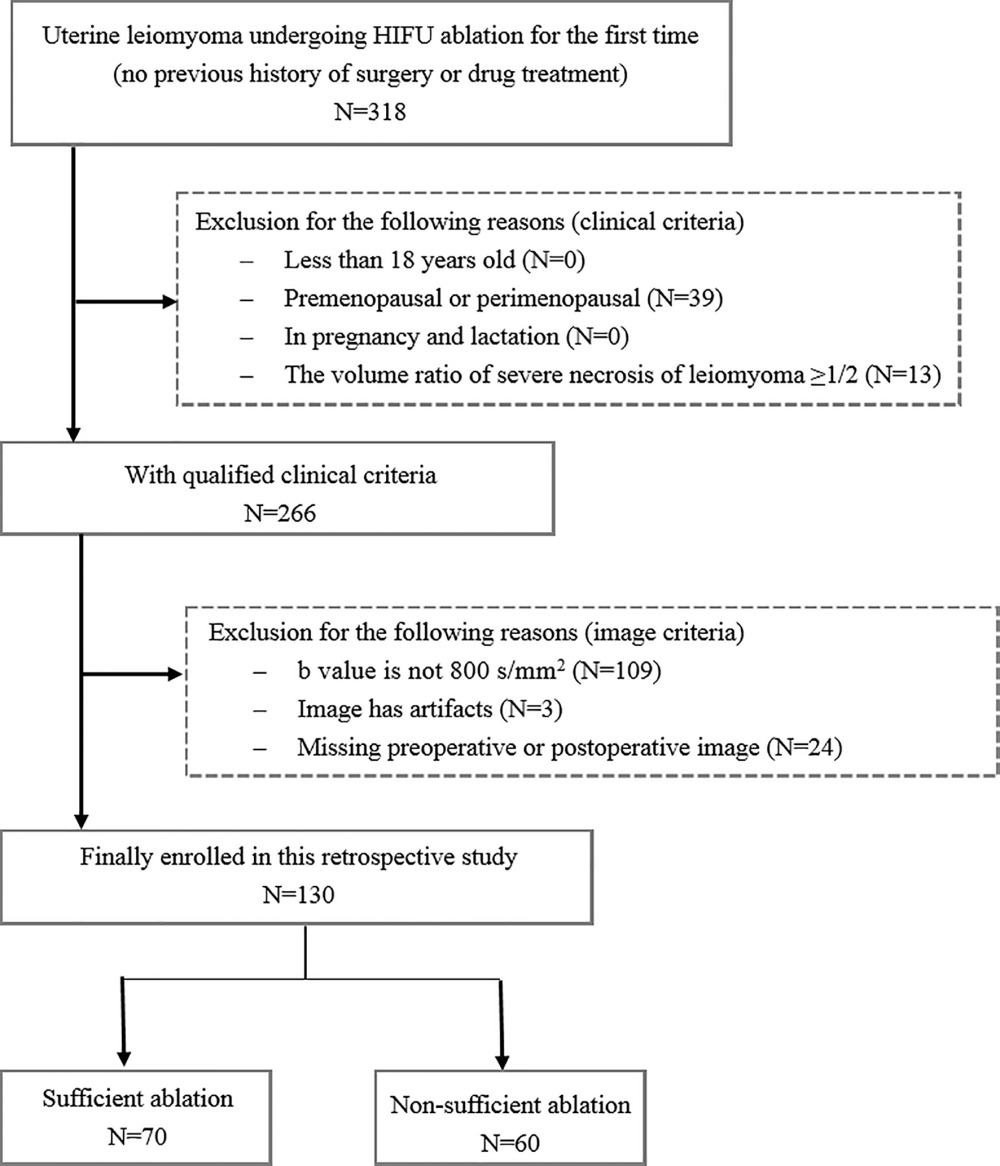
Patient recruitment pathway.

This single-center retrospective research was approved by the Institutional Review Board of the First Affiliated Hospital of Chongqing Medical University, and the patient consent was waived. From January 2013 to December 2018, 318 patients receiving HIFU ablation therapy for uterine leiomyomas were enrolled for this analysis. The inclusion criteria were as follows: (1) above 18 years of age, (2) premenopausal or perimenopausal, (3) no previous history of surgery or drug treatment, and (4) leiomyomas diameter ≥3 cm. The exclusion criteria were as follows: (1) patients have contraindications for MRI examination and contrast injection, (2) the volume ratio of severe necrosis of leiomyomas is ≥1/2, and (3) patients are in pregnancy and lactation. **Figure 2** shows the flowchart of patient enrollment and exclusion criteria. Finally, a total of 130 patients with uterine leiomyomas were included for the following analysis. According to the relationship between treatment outcome and HIFU ablation efficiency reported in the previous studies (19), the ablation rate of uterine leiomyomas was used to divide the patients into two groups: 70 uterine leiomyomas with sufficient ablation (ablation rate ≥70%) and 60 uterine leiomyomas with nonsufficient ablation (ablation rate <70%). The ablation rate was defined as the ratio between the nonperfused volume in the target uterine leiomyoma after HIFU ablation and the volume of original uterine leiomyoma before treatment. The calculation of ablation rate was implemented in a standard picture archiving and communication system (PACS) workstation (Carestream Health, Rochester NY) by outlining the target leiomyoma before treatment and the unenhanced areas of that after treatment in the contrast-enhanced images layer by layer.

### Multiparametric MRI Scanning Protocol

In this study, each patient underwent MRI examination using 3.0T system (Signa HDxt, GE Medical System) before and after HIFU ablation, respectively. The postoperative MRI examination was within 7 days after treatment. The imaging protocol included (1) fat-suppressed fast spin-echo T2WI imaging in the axial, coronal, and sagittal planes; (2) axial fast spin-echo T1-weighted (T1WI) imaging; (3) axial DWI with reconstruction of ADC map; and (4) dynamic contrast-enhanced (DCE) MRI. The parameters of T2WI imaging and DWI are presented in **Table 1**.

**Table 1:** Parameters for MRI sequences.

Parameter	T2WI	DWI
Scanning plane	Axial	Axial
TR/TE (ms)	4,380/106	4,000/62.9
Slice thickness (mm)	5	6
Slice gap (mm)	1.5	1.5
Field of view (cm)	28 × 22.4	38.0 × 45.8
Matrix	320 × 224	128 × 130
*b*-value (s/mm^2^)	N/A	800

N/A, not applicable.

### Image Segmentation

**Figure 3:**
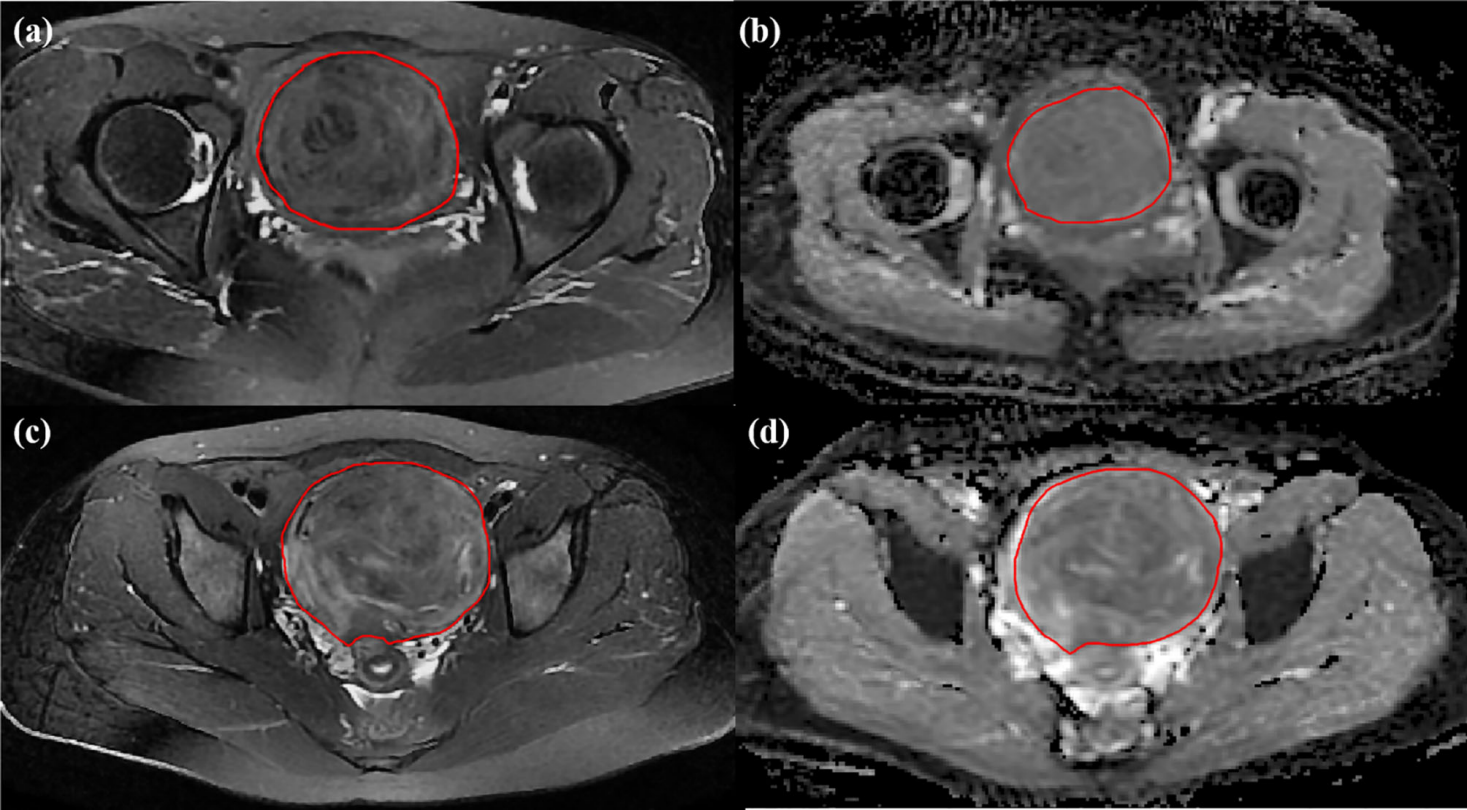
Two cases for delineating ROI. The preoperative MR images for uterine leiomyomas with sufficient ablation on **(A)** T2WI image and **(B)** ADC map from DWI and with nonsufficient ablation on **(C)** T2WI image and **(D)** ADC map from DWI.

All MRI images were exported from PACS in DICOM format. Two blinded abdominal radiologists with 7 and 12 years of experience in pelvic radiological imaging independently interpreted all MR images and manually determined the regions of interest (ROIs) by delineating the margin of leiomyoma using ITK-SNAP software (www. itksnap.org) in the axial plane, as shown in **Figure 3**. The ROIs of DWI with a *b*-value of 800 s/mm^2^ were delineated on corresponding apparent diffusion coefficient (ADC) maps. The principles of ROI sketching were as follows: (1) sketching layer by layer to form the 3D ROIs of the lesion; (2) including the cystic and necrotic area of the lesion; (3) sketching the maximum extent of the lesion as much as possible, if the tumor boundary is blurred.

### Radiomics Feature Extraction

In this study, a total of 972 candidate radiomics features were extracted from each of T2WI image and ADC map, which comprised first-order statistic features, intensity- and shape-based features, high-order textural features, and wavelet transform-based features. Textural features were divided into five categories such as gray-level co-occurrence matrix (GLCM), gray-level run-length matrix (GLRLM), gray-level size zone matrix (GLSZM), neighborhood gray-tone difference matrix (NGTDM), and gray-level dependence matrix (GLDM) (20, 21). Wavelet features are the transformed domain representations of the intensity and textural features, which were computed on the wavelet decomposition of the original image. Then, the radiomics feature set was performed z-score normalization. The PyRadiomics package (http://pyradiomics.readthedocs.io) implemented in Python (version 3.6) was used for radiomics feature extraction referring to the corresponding mathematical definitions (22).

### Feature Reproducibility Evaluation and Selection

Intraclass correlation coefficients (ICC) were used to evaluate the agreement and robustness of extracted features from the different ROIs in the same images between two observers. The reproducibility of radiomics features were evaluated by computing ICC prior to feature selection. An ICC value of more than 0.8 was considered to represent excellent consistency (23). The optimal characterization condition often means the minimal prediction error (24), so feature selection as an important factor for pattern classification plays an important role in the processing of high-dimensional radiomics features. The algorithms of recursive feature elimination (RFE), ReliefF, and least absolute shrinkage and selection operator (LASSO) were used for feature selection in this study (25, 26). Additionally, publicly available implementations were readily available for these methods, which increase the reusability of the results in this study.

### Machine Learning Model

For the prediction model development, four machine learning classifiers such as k-nearest neighbors (KNN), logistic regression (LR), random forest (RF), and support vector machine (SVM) were implemented in Python environment (version 3.6). The reason for choosing these classifiers was that they have been proven to rank at the top of prediction performance and commonly used in the related study.

### Performance Validation

To ensure robust and efficient prediction performance of model, the selective features by the different feature selection methods were used to construct models with these four machine learning algorithms one-by-one, and the analysis of performance comparison were performed by means of standard performance metrics including the area under the ROC curve (AUC), accuracy, sensitivity, and specificity in fivefold cross-validation. The most efficient combination of machine learning and feature selection method with the highest accuracy was determined as predictive model, which was used to predict the treatment outcome of HIFU ablation for uterine leiomyomas.

### Statistical Analysis

Continuous variables, expressed as mean value ± standard deviation or median with interquartile range as appropriate, were analyzed using Student’s *t*-test or Mann-Whitney *U*-test, respectively. ROC curves were calculated from all validation sets to show generalization. The comparisons of AUCs were accomplished using the DeLong nonparametric approach (27). Decision curve analysis was employed to evaluate the clinical usefulness of the radiomics model. Calibration curve along with the Hosmer-Lemeshow test was used to evaluate the similarity between the predicted and observed probabilities (28). Statistical analysis was performed with R software (version 3.6.1). A two-sided *p* < 0.05 was considered to represent statistically significant.

## Results

### Clinical Characteristics

The clinical and radiological characteristics of the patients are summarized in **Table 2**, and there were no significant differences between the primary and validation cohorts. Between the high and low ablation groups, there were no significant differences in the primary and validation cohorts in the volume, size, subtypes, and location of leiomyomas (*p* > 0.05, **Table 3**). A few radiological features were significantly different between two groups in the primary or validation cohorts, including T2 signal intensity, T2 signal homogeneity, and uterine position (**Table 3**).

**Table 2:** The comparison of demographic information and radiological image characteristics between primary and validation cohorts.

Characteristics	Primary cohort	Validation cohort	p-Value
*N*	104	26	
Ablation efficacy			
High (≥70%)	56	14	1.000^b^
Low (<70%)	48	12	
Age	38.32 ± 6.27	39.14 ± 6.59	0.211^a^
Volume (mm^3^)	91.54 (52.08–159.07)	79.12 (46.02–122.47)	0.322^c^
Size (mm)	56.85 (45.32–67.87)	49.45 (43.20–64.43)	0.618^c^
Type			
Submucous	5	2	0.481^b^
Myometrial	89	20	
Subserous	10	4	
Location			
Anterior wall	64	15	0.893^b^
Posterior wall	40	11	
T2 signal intensity			
Hyperintensity	40	9	0.892^b^
Hypointensity	64	17	
T2 signal homogeneity			
Homogeneous	14	7	0.133^b^
Inhomogeneous	90	19	
Uterine position			
Anteversion	73	15	0.385^b^
Retroversion	31	11	
Energy efficiency factor (J/mm^3^)	3.6 (1.6–7.1)	3.7 (1.8–6.9)	0.118^c^
Sonication time (s)	790 (380–1,360)	815 (405–1,420)	0.247^c^

a p-Values obtained using independent-sample t-test.

b p-Values obtained using Chi-squared test or Fisher’s exact test.

c p-Values obtained using Wilcoxon rank-sum test.

**Table 3:** Demographic information and radiological image characteristics between the high and low ablation groups.

Characteristics	Primary cohort		p-Value	Validation cohort		p-Value
	High ablation (≥70%)	Low ablation (<70%)		High ablation (≥70%)	Low ablation (<70%)	
*N*	56	48		14	12	
Age	38.76 ± 5.96	37.81 ± 6.65	0.442^a^	39.07 ± 5.86	38.58 ± 7.84	0.858^a^
Volume (mm^3^)	84.65 (50.6–152.9)	99.05 (56.52–180.10)	0.173^c^	73.9 (57.32–121.70)	88.15 (68.65–117.75)	0.311^c^
Size (mm)	53.15 (41.47–63.62)	59.65 (46.55–70.45)	0.202^c^	49.80 (35.40–52.75)	57.75 (48.67–71.85)	0.197^c^
Type						
Submucous	3	2	0.914^b^	1	1	0.791^b^
Myometrial	47	42		10	10	
Subserous	6	4		3	1	
Location						
Anterior wall	35	29	0.987^b^	6	9	0.209^b^
Posterior wall	21	19		8	3	
T2 signal intensity						
Hyperintensity	19	21	0.409^b^	13	4	0.003^b^
Hypointensity	37	27		1	8	
T2 signal homogeneity						
Homogeneous	10	4	0.258^b^	7	1	0.030^b^
Inhomogeneous	46	44		7	11	
Uterine position						
Anteversion	46	27	0.007^b^	6	9	0.209^b^
Retroversion	10	21		8	3	

a p-Values obtained using independent-sample t-test.

b p-Values obtained using Chi-squared test or Fisher’s exact test.

c p-Values obtained using Wilcoxon rank-sum test.

### Predictive Performance Comparison of Machine Learning Models

**Figure 4:**
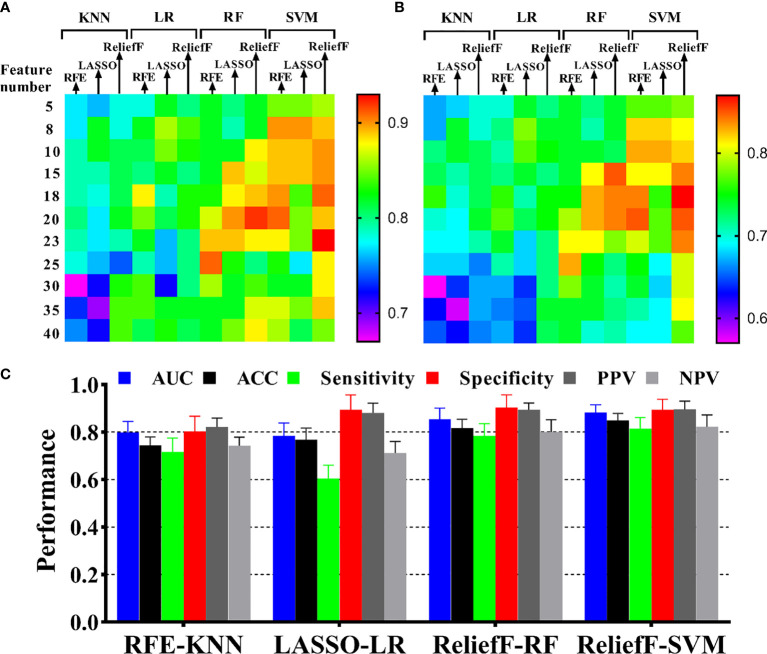
Performance of HIFU ablation prediction with different machine learning methods. The heatmaps show the AUCs of model with four classifiers and three feature selection methods in different feature numbers for **(A)** the primary cohort and **(B)** the validation cohort. The annotation of the heatmap (located to the right of the entire image) illustrates that red or yellow represents a high AUC and pink or blue represents a low AUC. **(C)** Model performance presentation for the four optimal combinations of feature selection and machine learning in the validation cohort.

**Figure 5:**
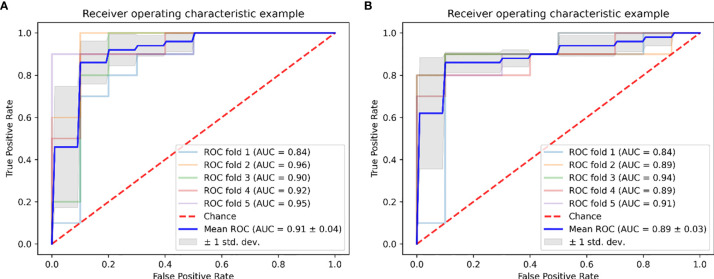
Graph shows receiver operating characteristic curves of ReliefF-SVM model for outcome prediction of HIFU treatment in **(A)** the primary and **(B)** validation cohorts.

In the predictive model building, the performance of different models was investigated, where four machine learning classifiers and three feature selection algorithms were tested, and the corresponding heatmaps of AUCs in the primary and validation cohorts using multiparameter MRI were shown in **Figure 4**. For the different feature selection methods, the most suitable machine learning classifier is individualized. For RFE and LASSO, KNN, and LR models work better, respectively. ReliefF algorithm is more suitable to RF and SVM. The detail predictive performance metrics of the four machine learning models in the primary and validation cohorts were shown in **Figure 4C** and **Table 4**. The comparison result indicated SVM and RF outperformed LR and KNN significantly (all *p*-values <0.05), when the number of radiomics features ranged from 15 to 18 and the predictive performance of machine leaning models was optimal. The ReliefF-SVM model showed the best predictive performance with an AUC of 0.911 and accuracy of 0.884 in the primary cohort and an AUC of 0.887 and accuracy of 0.849 in the validation cohort (**Figure 5**), followed by ReliefF-RF model which yielded an AUC of 0.875 and accuracy of 0.851 in the primary cohort and an AUC of 0.854 and accuracy of 0.817 in the validation cohort (**Table 4**). The result of the DeLong test suggested that the prediction performance of the ReliefF-SVM model was significantly better than that of the ReliefF-RF model in the primary and validation cohorts (*p* = 0.021 and 0.044, respectively).

**Table 4:** The best performance of four radiomics models in the primary and validation cohorts.

Classifier	*N*	Cohort	AUC [95% CI]	Accuracy	Sensitivity	Specificity	PPV	NPV
RFE-KNN	18	Primary	0.798 [0.754–0.836]	0.764	0.723	0.816	0.863	0.762
		Validation	0.762 [0.721–0.807]	0.744	0.716	0.802	0.822	0.743
LASSO-LR	8	Primary	0.861 [0.824–0.922]	0.833	0.775	0.917	0.885	0.790
		Validation	0.784 [0.755–0.834]	0.769	0.605	0.894	0.881	0.712
ReliefF–RF	15	Primary	0.875 [0.829–0.933]	0.851	0.809	0.892	0.878	0.830
		Validation	0.854 [0.816-0.907]	0.817	0.784	0.904	0.894	0.801
ReliefF-SVM	18	Primary	0.911 [0.854–0.973]	0.884	0.857	0.921	0.918	0.853
		Validation	0.887 [0.848–0.939]	0.849	0.814	0.896	0.903	0.823

### Performance of Sequences

Furthermore, we have investigated whether the radiomics features extracted from multiple sequences can better predict the therapeutic response of uterine leiomyoma to HIFU ablation using the ReliefF-SVM model. For single sequence, the SVM classifier using the DWI sequence yielded an AUC of 0.831 (95% CI = 0.740–0.922) in the primary cohort and an AUC of 0.789 (95% CI = 0.689–0.889) in the validation cohort, while it yielded an AUC of 0.863 (95% CI = 0.796–0.929) in the primary cohort, and an AUC of 0.822 (95% CI = 0.755–0.888) in the validation cohort using the T2WI sequence. The performance of T2WI sequence is better than that of DWI sequence. However, when use the combination of T2WI and DWI sequences, the SVM classifier yielded the highest AUC of 0.911 (95% CI = 0.854–0.973) in the primary cohort and an AUC of 0.887 (95% CI = 0.848–0.939) in the validation cohort. In general, for the multiparametric MRI, the SVM classifier with ReliefF algorithm had the best performance.

### Clinical Usefulness

**Figure 6:**
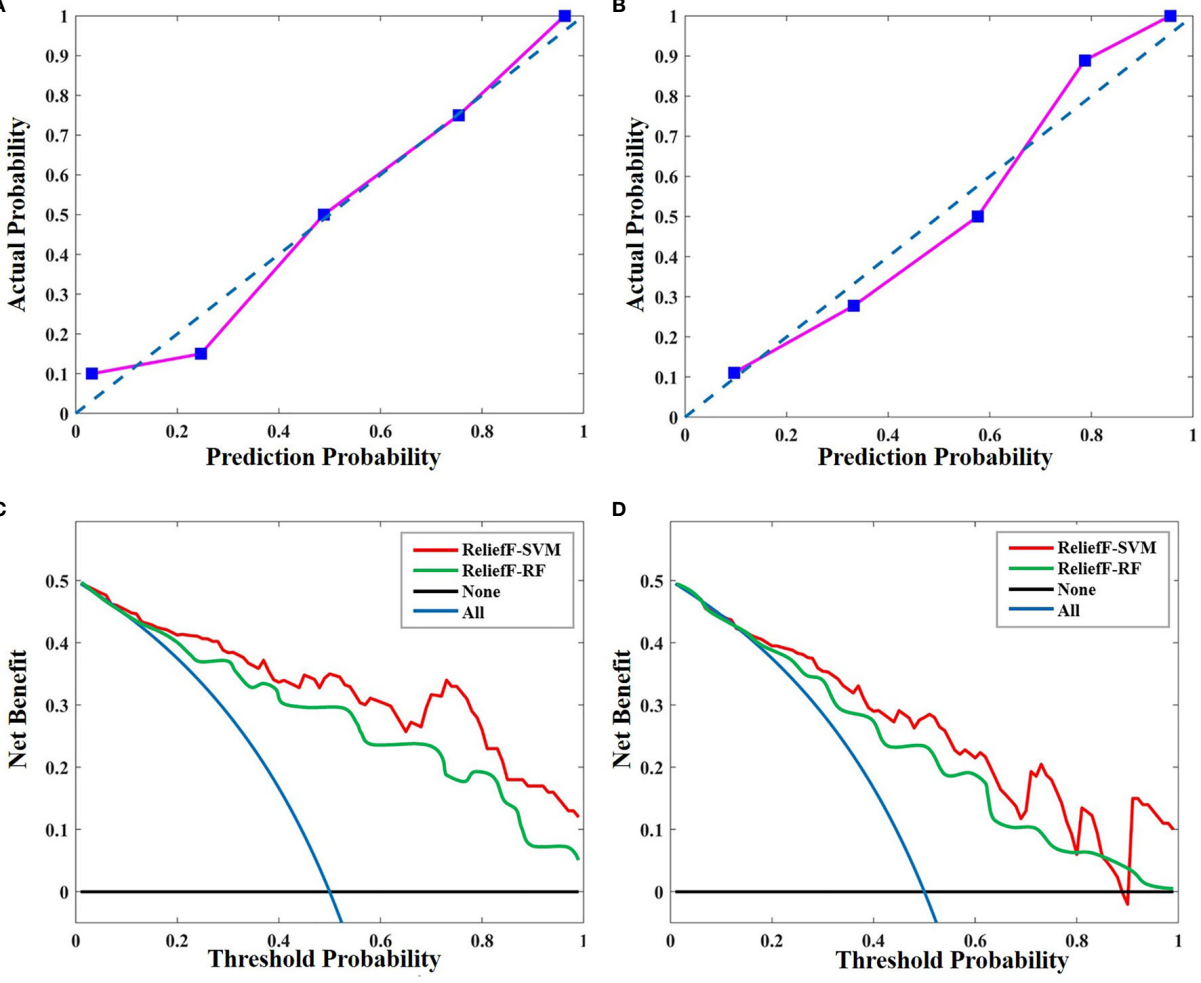
Calibration curves of the radiomics model in **(A)** the primary and **(B)** validation cohorts. Calibration curves depict the calibration of ReliefF-SVM model in terms of the agreement between the predicted probability and actual outcomes. The *y*-axis represents the actual rate of HIFU probability. The *x*-axis represents the predicted probability. The diagonal blue line represents a perfect prediction by an ideal model. The pink line represents the performance of the radiomics model, where a closer fit to the diagonal blue line represents a better prediction. Decision curve analysis for the ReliefF-SVM and ReliefF-RF models in **(C)** the primary and **(D)** validation cohorts. The *y*-axis measures the net benefit. The red and green lines represent the radiomics model. The blue line represents the assumption that all patients received high HIFU ablation. The black line represents the assumption that no patients received high HIFU ablation.

The calibration curves of the radiomics model for the therapeutic response of uterine leiomyoma to HIFU ablation demonstrated good agreement between observation and prediction in both the primary dataset (**Figure 6A**) and validation dataset (**Figure 6B**). The Hosmer–Lemeshow test revealed no statistically significant departure from a perfect fit (*p* = 0.632 and *p* = 0.498, respectively). Then, the decision curves showed that the ReliefF-SVM model provided a higher overall net benefit for predicting clinical outcome for HIFU treatment than the ReliefF-RF model in the primary and validation cohorts (**Figures 6C, D****)**. The threshold probability was within the range of 0.24 to 0.86. This indicates that the radiomics machine learning model is clinically useful and has favorable performance in the outcome prediction of HIFU ablation of uterine leiomyomas.

## Discussion

HIFU as a noninvasive therapeutic technique, can selectively produce typical coagulation necrosis at a precise focal point within leiomyomas lesions. It has several attractive advantages including noninvasion, nonsurgical treatment, lower risk, no ionizing radiation, and faster recovery time (29, 30), when compared with the other noninvasive or minimally invasive therapeutic methods such as vascular embolization, radiofrequency ablation, cryotherapy, and targeted radiotherapy (31).

In this study, we developed 12 machine learning models for the preoperative outcome prediction of HIFU ablation for uterine leiomyomas using multiparametric MRI radiomics features. The ReliefF-SVM-based radiomics machine learning model showed favorable predictive performance in the current data cohort, demonstrating the potential to predict individual treatment outcome for the patients with uterine leiomyomas.

This study investigated different combinations of the frequently used feature selection algorithms and machine learning classifiers for the construction of outcome prediction models and performed the performance comparison of different combinations. The result indicated that these machine learning models achieved favorable performance in predicting the clinical success for HIFU treatment, and the best performance was found in the combination of SVM classifier and ReliefF algorithm. This showed consistency with previous studies where machine learning exhibits a potential in clinical application such as preoperative gastrointestinal stromal tumor prediction, Parkinson’s disease, and breast lesion classification (32, 33).

Preoperative prediction of treatment outcome is essential before HIFU intervention and is also an important factor to determine individual therapeutic schedule for the patients with uterine leiomyomas. The previous studies have investigated the relationship between some qualitative radiological indicators and the difficulty level of HIFU ablation of uterine leiomyomas. The results revealed the enhancement pattern of leiomyoma lesion on T1WI image, and the signal intensity of that on T2WI image were related to the HIFU ablation efficiency (34–37). Furthermore, multivariate regression model was proposed to predict the difficulty level of HIFU ablation by estimating the energy efficiency factor and sonication time. However, there is currently no accurate machine learning model for predicting the therapeutic response of uterine leiomyomas to HIFU ablation using the quantitative radiomics characteristics on unenhanced MR image. To our knowledge, this is the first study that uses radiomics analysis and machine learning model for the preoperative prediction of clinical outcome for HIFU ablation.

Our study revealed that the quantitative radiomics features from the preoperative MR images could effectively describe the degree of difficulty in HIFU ablation of uterine leiomyomas. This result is supported by literature (36, 38) which also demonstrated that the blood supply of uterine fibroids, tissue structure of fibroids, and the size of fibroids are important factors influencing the HIFU ablation efficiency (10). Some studies have shown that uterine leiomyomas displaying the mixed hyperintensity on T1WI image with significant enhancement or the hyperintensity on T2WI image had worse therapeutic response of uterine leiomyoma to HIFU ablation (36, 39). This demonstrates that radiomics analysis has potential in the preoperative prediction of treatment outcome for leiomyoma HIFU ablation, because it could feasibly be used to quantify the different textures that are regarded as different patterns of hyperintensity or hypointensity in MR images.

In this study, we investigated the feasibility of applying radiomics and machine learning to predict the efficiency of HIFU ablation on conventional unenhanced MR images. The ability to predict preoperatively HIFU treatment outcome on T2WI image and ADC map can potentially minimize the need for contrast agent administration as well as avoid the contrast agent adverse events.

There were several limitations in this study. Firstly, the data were obtained from a single institution, which lacked the validation data from different image acquisition protocols for generalization. Secondly, the conventional clinical parameters were not involved in the prediction model, and we only investigated two routine MR sequences such as T2WI and DWI sequences. In the future, other advanced sequences such as diffusion tensor imaging, perfusion-weighted imaging, and MR spectroscopy combined with clinical parameters should be involved. Thirdly, segmentation of uterine leiomyomas was performed manually, because of the challenges encountered in automatic VOI labeling and complex anatomical structure delineation in abdominal MR image.

In conclusion, this study developed a multiparametric MRI radiomics-based machine learning model for the preoperative prediction of HIFU treatment outcome for uterine leiomyomas by the comparison of different machine learning models. The ReliefF-SVM model showed favorable performance in predicting clinical outcome of HIFU ablation in the current data. If the model will be further developed and validated, it may help clinicians better screen patients who can most benefit from HIFU therapy and provide a reference for treatment decision-making.

## Data Availability Statement

The data analyzed in this study is subject to the following licenses/restrictions: The datasets for this article are not publicly available as it is private data that belongs to The First Affiliated Hospital of Chongqing Medical University. Requests to access the datasets should be directed to corresponding author. Requests to access these datasets should be directed to fajinlv@163.com.

## Ethics Statement

The studies involving human participants were reviewed and approved by Institutional Review Board of The First Affiliated Hospital of Chongqing Medical University. Written informed consent for participation was not required for this study in accordance with the national legislation and the institutional requirements.

## Author Contributions

YZ performed the experimental design, manuscript writing, data analysis, and prepared tables and figures. LC performed the data gathering. ML, JW and RY aided with analysis of the data. FL supervised the experimental design, data analysis, manuscript writing and editing. All authors contributed to the article and approved the submitted version.

## Funding

This study was supported by the Natural Science Foundation of Chongqing (cstc2019jcyj-msxmX0395) and the Intelligent Medicine Research Project of Chongqing Medical University (ZHYX202009).

## Conflict of Interest

The authors declare that the research was conducted in the absence of any commercial or financial relationships that could be construed as a potential conflict of interest.

## Publisher’s Note

All claims expressed in this article are solely those of the authors and do not necessarily represent those of their affiliated organizations, or those of the publisher, the editors and the reviewers. Any product that may be evaluated in this article, or claim that may be made by its manufacturer, is not guaranteed or endorsed by the publisher.
